# Back to front: cerebellar connections and interactions with the prefrontal cortex

**DOI:** 10.3389/fnsys.2014.00004

**Published:** 2014-02-04

**Authors:** Thomas C. Watson, Nadine Becker, Richard Apps, Matthew W. Jones

**Affiliations:** School of Physiology and Pharmacology, University of BristolBristol, UK

**Keywords:** cerebellum, fastigial nucleus, prefrontal cortex, prelimbic cortex, theta, coherence

## Abstract

Although recent neuroanatomical evidence has demonstrated closed-loop connectivity between prefrontal cortex and the cerebellum, the physiology of cerebello-cerebral circuits and the extent to which cerebellar output modulates neuronal activity in neocortex during behavior remain relatively unexplored. We show that electrical stimulation of the contralateral cerebellar fastigial nucleus (FN) in awake, behaving rats evokes distinct local field potential (LFP) responses (onset latency ~13 ms) in the prelimbic (PrL) subdivision of the medial prefrontal cortex. Trains of FN stimulation evoke heterogeneous patterns of response in putative pyramidal cells in frontal and prefrontal regions in both urethane-anesthetized and awake, behaving rats. However, the majority of cells showed decreased firing rates during stimulation and subsequent rebound increases; more than 90% of cells showed significant changes in response. Simultaneous recording of on-going LFP activity from FN and PrL while rats were at rest or actively exploring an open field arena revealed significant network coherence restricted to the theta frequency range (5–10 Hz). Granger causality analysis indicated that this coherence was significantly directed from cerebellum to PrL during active locomotion. Our results demonstrate the presence of a cerebello-prefrontal pathway in rat and reveal behaviorally dependent coordinated network activity between the two structures, which could facilitate transfer of sensorimotor information into ongoing neocortical processing during goal directed behaviors.

## Introduction

An increasing number of studies advocate the view that cerebellar contributions to behavior are not confined to motor control but also extend to cognitive function (e.g., Stoodley and Schmahmann, 2010). Consistent with this, convergent evidence from clinical, neuroimaging and anatomical tracing studies in primates suggests that the cerebellum forms “closed-loop” connections with neocortical brain regions including the prefrontal cortex (Middleton and Strick, 1994, 2001; Kelly and Strick, 2003; Schmahmann, 2004; Allen et al., 2005; Krienen and Buckner, 2009; Strick et al., 2009; O'Reilly et al., 2010; Stoodley and Schmahmann, 2010; Buckner et al., 2011; Stoodley, 2012). These anatomical connections provide the neural basis through which cerebellar contributions to neocortical processing may occur, enabling integration of sensorimotor information across hind- and fore-brain.

The understanding of such distributed networks may be especially pertinent given that abnormal prefrontal-cerebellar interactions are implicated in disorders such as autism and schizophrenia (Andreasen et al., 1996; Andreasen and Pierson, 2008; Fatemi et al., 2012). In particular, imaging studies frequently report abnormalities of the cerebellar vermis in schizophrenia (e.g., Okugawa et al., 2007; Lawyer et al., 2009; Henze et al., 2011) and direct electrical and transcranial magnetic stimulation of the vermis has shown some efficacy in treating the cognitive and emotional symptoms of the disease (Heath, 1977; Demirtas-Tatlidede et al., 2010).

Although the presence of a cerebello-cortical reciprocal network has not been demonstrated in non-primates, electrophysiological and amperometric studies have highlighted, respectively, the existence of a prefrontal-olivo-cerebellar pathway in anesthetized rats (specifically to vermal lobule VII; Watson et al., 2009), and modulation of prefrontal dopamine release following cerebellar stimulation in anesthetized mice (Mittleman et al., 2008). Anatomical data also suggest the existence of disynaptic fronto-cerebellar connectivity in rat (Suzuki et al., 2012) and preliminary data obtained in mouse suggest a neural connection exists between the cerebellar nuclei and prefrontal cortex (Arguello et al., 2012). Recent evidence has also highlighted the importance of cerebellar plasticity in goal-directed behavior and spatial navigation in mice (Burguière et al., 2010; Rochefort et al., 2011). Together, these studies suggest the basis of a rodent prefrontal-cerebellar network reminiscent of that described anatomically in primates (Middleton and Strick, 2001; Kelly and Strick, 2003).

While the neuroanatomical basis of non-human primate prefrontal-cerebellar networks is relatively well established, and becoming clearer in rodents (see above), scant information is available on the dynamic physiological interactions between the two structures, particularly during behavior. This is of importance given that temporally organized, large-scale, distributed networks are thought to be fundamental to information processing as reflected in frequency specific, coherent local field potential (LFP) oscillations (Gray, 1994; Varela et al., 2001; Fries, 2005). Indeed, coherent cerebro-cerebellar oscillations have been previously observed, across frequencies ranging from 1 to 40 Hz, in both anesthetized and awake, behaving animals (O'Connor et al., 2002; Courtemanche and Lamarre, 2005; Soteropoulos and Baker, 2006; Ros et al., 2009; Rowland et al., 2010). Nevertheless, it remains unknown whether prefrontal cortical activity also synchronizes with the cerebellum.

We therefore sought to address the basis and nature of cerebello-prefrontal interactions in rat by: (a) using electrophysiological mapping techniques to study connectivity between the cerebellar vermal output nucleus, fastigius and neocortical regions, including the PrL; (b) exploring the possibility that cerebellar stimulation may modulate ongoing firing patterns in neocortical regions; and (c) examining the coordination of LFP activity within this network.

## Methods

All experimental procedures were carried out in accordance with the UK Animals (Scientific Procedures) Act 1986 and were approved by the University of Bristol institutional animal licence advisory group. A total of 13 adult rats were used in two experimental groups: non-recovery electrophysiology (8 Wistar rats, weight 280–380 g, Harlan, UK) and chronic, recovery electrophysiology (4 Long-Evans and 1 Wistar, weight 340–440 g, Harlan, UK). Strain-related differences were not apparent in any of the results.

### Electrophysiology in anesthetized rats

Rats were anesthetized with urethane (1.5 g/kg intraperitoneal injection) then placed in a stereotaxic frame (David Kopf instruments, Tujunga, CA) and secured with atraumatic ear bars coated with a topical local anesthetic (Xylocaine, Astra Pharmaceuticals, Kings Langley, UK). Occasionally a supplementary dose of urethane was given (10 % of original dose) to maintain surgical levels of anesthesia, as evidenced by the absence of limb withdrawal and corneal reflexes and lack of whisking. Core body temperature was maintained at 36–38°C through the use of a homoeothermic blanket (Harvard apparatus, Massachusetts, USA). Craniotomies were made over the frontal cortex (+3.2 mm, +0.6 mm from bregma) and cerebellum (−11.5 mm, +0.8 mm from bregma).

Cortical recordings were made using a Cheetah 32 system (Neuralynx, Montana, USA), with extracellular action potentials (sampled at 32 kHz and filtered between 0.6–6 kHz) recorded differentially using a local reference placed in a proximal cortical region in which spiking activity was absent. Typically, arrays of six extracellular tetrode recording electrodes were positioned on the surface of secondary motor cortex (M2) whilst a bipolar stimulating electrode (SNE-100X, interpolar distance of 0.5 mm, Rhodes Electromedical) was targeted toward the contralateral cerebellar fastigial nucleus (FN, 4.5 mm from surface of brain) and used to deliver trains of stimuli at 0.03 Hz (intensity of 100 μA and frequency of 100 Hz; cf. Mittleman et al., 2008). Extracellular responses to the cerebellar stimulation were recorded at two depths within the frontal cortex: firstly in superficial regions (M2/anterior cingulate border; 1.3–2 mm ventral from brain surface) and once again when the tetrodes had been lowered to their final position in the PrL region (2.6–3 mm ventral from brain surface). On average, we recorded 2.4 ± 0.2 well-isolated units per tetrode in anesthetized animals; continuous LFP was not recorded in these anesthetized preparations.

### Electrophysiology in chronically implanted rats

Rats were implanted with up to 8 tetrode recording electrodes into the left frontal cortex (+3.2 mm, +0.6 mm from bregma) and 1 bipolar stimulating/recording electrode into the contralateral FN (−11.5 mm, +0.8 mm from bregma; interpolar distance of 0.5 mm) under sodium pentobarbital recovery anesthesia. In one animal, tetrodes were implanted in both cerebellum and frontal cortex at the same coordinates as given above. Following surgery, the independently moveable tetrodes were lowered into the PrL subdivision of the prefrontal cortex (~2.6–3 mm ventral from brain surface) over the course of 1 week. Differential recordings were made using a Digital Lynx system (Neuralynx, Montana, USA) with a local reference placed in a proximal cortical region without spiking activity (2.4–2.7 mm below the pial surface for prefrontal recordings). On average we recorded 2.2 ± 0.6 units per tetrode in the chronically implanted rats.

**Figure 1:**
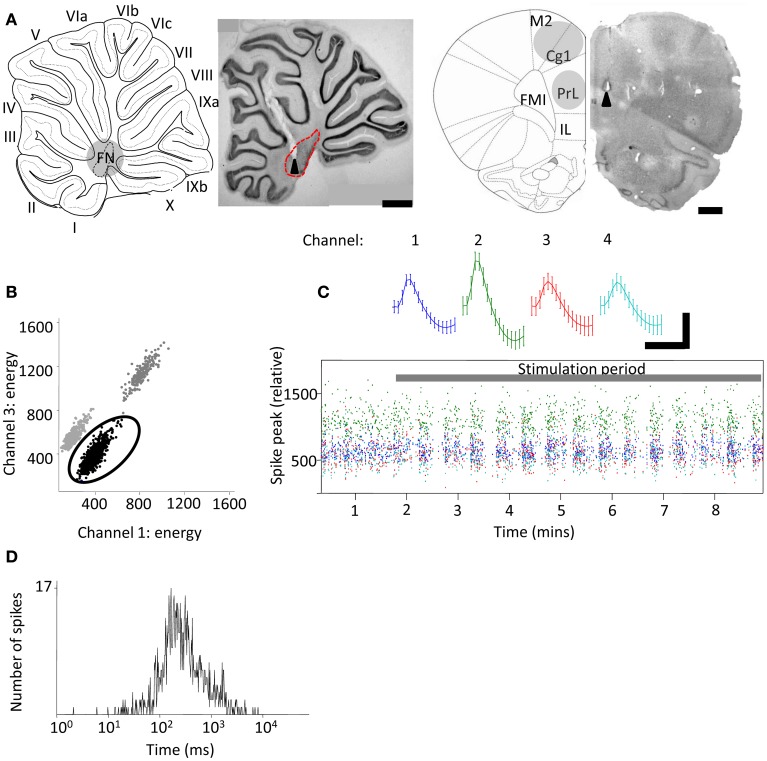
**Extracellular tetrode recordings of single-unit PrL activity during cerebellar stimulation**. **(A)** Grouped schematic (shaded gray areas, adapted from Paxinos and Watson, 2006) and representative micrographs of neutral red-stained 50 μm saggital and transverse brain slices showing respectively the sites of electrolytic lesions (arrowheads) in the cerebellum (left,) and PrL, right). Dashed lines indicate outline of fastigial nucleus and overlying cerebellar cortical lobules are numbered; scale bars, 1 mm. M2, supplementary motor cortex; Cg1, cingulate cortex; IL, infralimbic cortex. FMI, forceps minor of the corpus callosum. **(B)** Three clusters of action potentials spread along the axes of relative energy recorded on two channels of a tetrode in PrL. The properties of the black cluster (circled) are shown in **(C,D)**. **(C)** Mean waveform recorded on color-coded channels of the tetrode (top) showing stable relative spike amplitudes throughout one experiment (bottom) (scale bar, 0.05 mV; 0.7 ms). **(D)**, Distribution of interspike intervals (ISI) for all spikes fired by the unit in the experimental session.

Cerebellar LFP recordings were made through either previously implanted bipolar electrodes positioned in the cerebellum, with overlying skull screws serving as the reference point or in one case with tetrodes, which were referenced locally to a tetrode without spiking activity. LFP signals were sampled at 2 KHz and filtered between 0.1 and 475 Hz. Extracellular action potentials were sampled and filtered as for the acute, non-recovery experiments and recording channels were grounded to two screws in the skull overlying the cerebellum. In some cases electrodes were coated in DiI (1,1′-dioctadecyl-3,3,3′,3′-tetramethylindocarbocyanine perchlorate; Molecular Probes, Invitrogen, UK) prior to implantation, which, in addition to electrolytic lesioning, was used to help establish electrode tip positions at the end of each experiment (see Figure 1).

### Chronic recording and stimulation protocols

Evoked field potentials and single unit responses were recorded in the M2/anterior cingulate (Cg1) and PrL regions whilst animals were in a rest box, which consisted of an elevated platform (20 cm diameter) inside a wooden box (45 × 45 × 100 cm). In all rest box experiments animal movement was monitored continuously by video. For field potential experiments, stimulation parameters consisted of a triplet burst of 3 pulses (0.1 ms pulse duration, 3 ms inter-pulse interval) delivered once every 2 s. The mean stimulation intensity required to evoke reliably detectable field potentials was 300 ± 115 μA (range 100 μA to 500 μA; *n* = 4). For single unit experiments, trains of stimuli (100 Hz, 100 stimuli, 1 s duration, mean stimulus intensity 80 ± 20 μA, range 40 μA to 100 μA; *n* = 3) were delivered to FN every 5 s. These stimulus parameters have previously been shown to drive cerebellar nuclear output (Bagnall et al., 2009). For experiments in which recordings were made from both cerebellum and frontal cortex (*n* = 5), rat location was video tracked in the open field (a 1 m diameter circular arena) via light-emitting diodes attached to a powered headstage (Cheetah software; Neuralynx, Montana, USA).

### Data analysis

All data were processed in Matlab (Mathworks, USA) unless stated otherwise. LFP and single unit data were sorted based upon running speed (derived from video tracking data). For open field, a thresholding algorithm extracted stretches of LFP that fell within periods of active locomotion, defined as a z-score normalized running speed of greater than 0. These LFP sections were then used for subsequent analysis. For rest box recordings, LFP was selected when the rats were in a state of quiet wakefulness characterized by minimal locomotion and absence of frontal cortical sleep-spindle activity. Multitaper spectral analyses were performed using the Chronux toolbox (Bokil et al., 2010). Directed coherence—which uses autoregressive models of two LFP signals to estimate which signal best predicts the other—was calculated using custom scripts described and published elsewhere (e.g., Baker et al., 2006; Williams et al., 2009). Single units were manually isolated off-line (Figures 1B,C) using clustering software (MClust3.5; A.D.Redish, available at http://redishlab.neuroscience.umn.edu/MClust/MClust.html); inclusion criteria were set to isolation distance >15.0 and L-ratio <0.35 (cf. Harris et al., 2001). Putative pyramidal cells were classified on the basis of spike width, waveform and mean firing rate (Jung et al., 1998). Cross-correlograms were computed in Matlab and spike trains shuffle-corrected across trials then normalized by the number of spikes. Autocorrelograms were constructed in the same manner and normalized by the number of spikes.

Peri-stimulus histogram (PSTH) plots were calculated for 2 s pre- and 5 s post-stimulation epochs with mean baseline firing rate calculated from the pre-stimulus period. Firing rates were computed in 100 ms bins ± bootstrapped error estimate. Trial-averaged rate was calculated and smoothed by a Gaussian kernel. The standard deviation of the kernel was set to 0.1 s. Significance in firing rates was determined with a random permutation test performed with a minimum of 10,000 randomizations (cf. Hagan et al., 2012). Significance was assumed when mean ± bootstrapped error estimate was above/below the pre-stimulation baseline firing rate. Cell response characteristics were calculated using automated Matlab scripts and compared using χ^2^-tests and One-Way ANOVA with *post-hoc* Tukey's Multiple Comparison Test.

**Figure 2:**
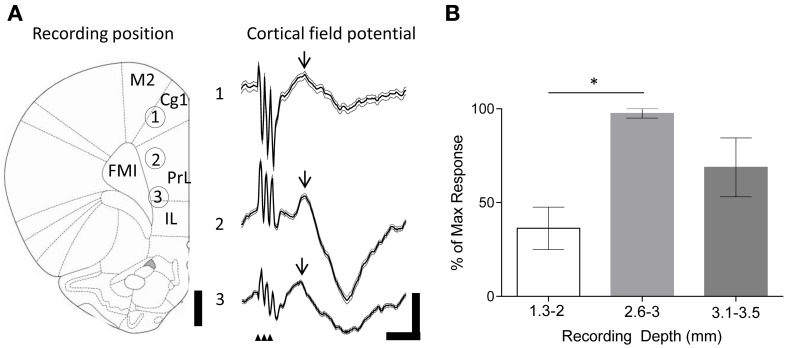
**Evoked field potentials in the frontal cortex following FN stimulation in behaving rats**. **(A)** Example experiment illustrating averaged (thick black line) field potentials (72 trials) recorded from tetrodes at different depths in the frontal cortex following stimulation of the FN (recording positions indicated by numbers on rat brain schematic adapted from Paxinos and Watson (2006); small arrowheads indicate timing of FN stimulation artefacts; M2, supplementary motor cortex; Cg1, cingulate cortex; IL, infralimbic cortex. FMI, forceps minor of the corpus callosum); scale bars, 1 mm and 0.1 mV, 20 ms, respectively. Thin gray lines idicates s.e.m. Arrow indicates field potential peak. **(B)** Grouped field potential peak-to-trough amplitudes expressed as a percentage of the maximal reponse size at superficial (1.3–2 mm) and intermediate (2.6–3 mm) and ventral (3.1–3.5 mm) recording positions (^*^P < 0.05; One-Way ANOVA with Tukey's multiple comparison test; *n* = 4; each data point calculated from 72 trials per animal).

Evoked field potential data were taken from a single tetrode channel and averages created in Spike2 software (Cambridge Electronic Design, UK). Evoked field potential onset latencies were measured from the time of the third and final stimulus of stimulation bursts to avoid contamination by stimulus artefacts (since total burst duration was 6 ms; see Figure 2A). Since absolute field potential amplitudes varied between animals, results were normalized and expressed as a percentage of the maximal response size. Response averages were then compared using One-Way ANOVA with *post-hoc* Tukey's Multiple Comparison Test. Data are presented as mean ± s.e.m. unless stated otherwise.

## Results

### Cerebello-prefrontal connectivity during quiet rest

We first examined the effects of FN stimulation on LFP in the frontal cortex in rats during quiet rest. In three out of four experiments FN stimulation evoked the largest field potentials at depths of 2.6–3 mm from the cortical surface. This depth range corresponds to PrL (see Figure 2). The evoked field potentials had an average onset latency of 13.1 ± 1.1 ms and peak-to-trough amplitude of 0.23 ± 0.12 mV. By comparison, superficially positioned tetrodes recorded evoked field potentials that were ~60% smaller in size (0.09 ± 0.03 mV; onset latency of 13.2 ± 1.5 ms Figure 2B). Recordings from ventral PrL (depth 3.1–3.5 mm) revealed evoked field potentials that were ~30% smaller than those recorded at 2.6–3 mm (0.16 ± 0.04 mV; onset latency of 13.2 ± 1.6 ms). Although changes in size of LFPs in the cerebral cortex should be interpreted with caution because current source-sink relationships are complex, nonetheless, the systematic variation in field potential amplitude found in the present study raises the possibility that this reflects a preferential physiological connectivity between FN and PrL compared to other areas of frontal cortex that were sampled.

### Modulation of prefrontal firing following FN stimulation

Following the discovery of cerebellar-prefrontal connectivity at the field potential level, we next sought to examine whether FN stimulation could modulate the ongoing firing patterns of individual frontal cortical neurons.

**Figure 3:**
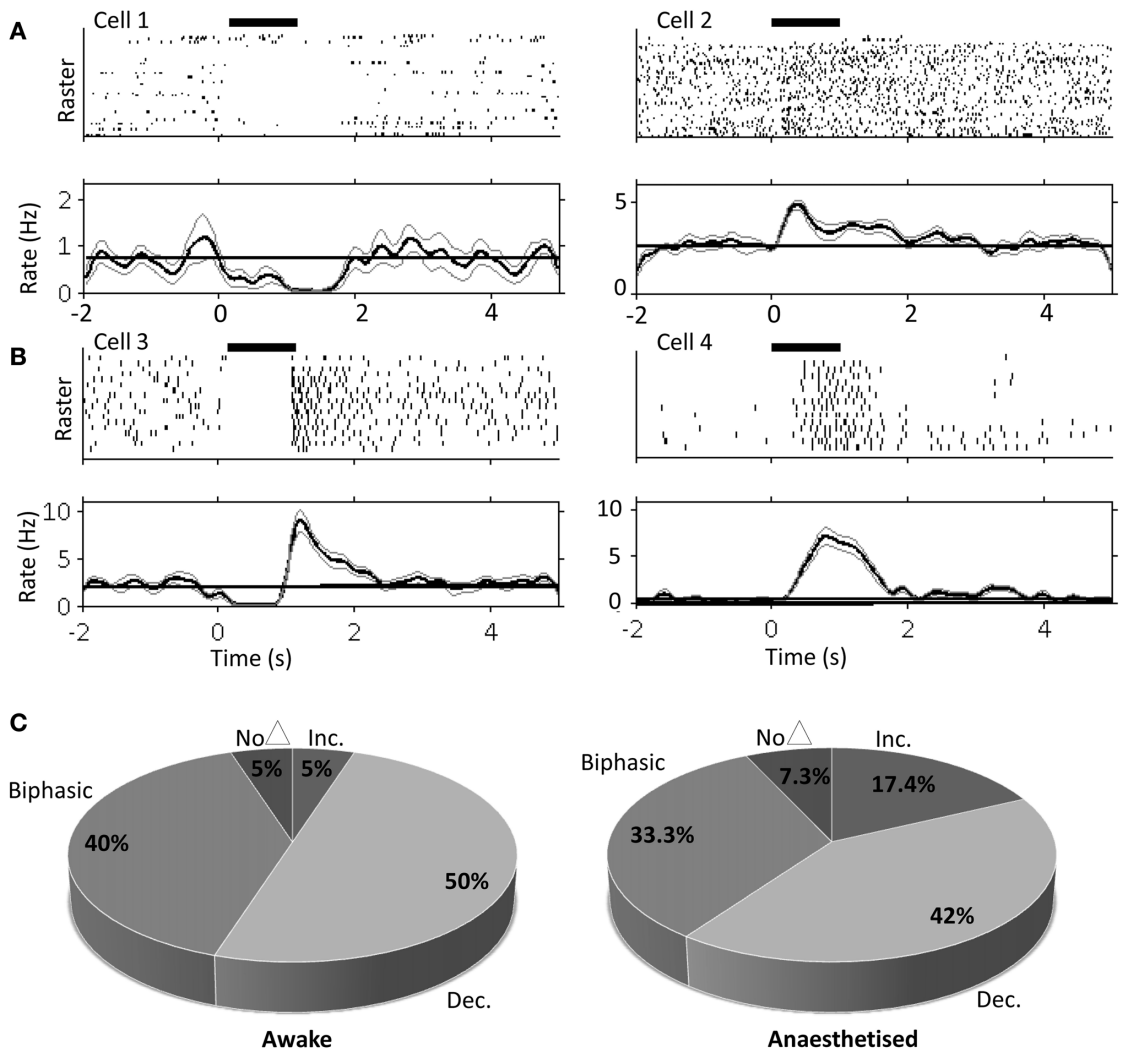
**Single unit PrL responses following FN stimulation in awake and urethane anesthetized rats**. Raster and peri-stimulus rate plots for example cells recorded in PrL in the awake animal, cells 1 and 2 **(A)** and urethane anesthetized animal, cells 3 and 4 **(B)**. Horizontal black bar indicates duration of stimulation (100 μA; 100 Hz; 1s duration); bold line indicates instantaneous mean firing rate; gray lines indicate bootstrapped error estimate; horizontal line indicates mean baseline firing rate prestimulation; bin size, 100 ms; 17 trials for anesthetized and 33 trials for awake experiments. **(C)** Quantification of PrL cell firing patterns following FN stimulation in awake (*n* = 20 putative pyramidal cells from 3 animals) and anesthetized rats (*n* = 69 putative pyramidal cells from 8 animals).

The large projection neurons in the cerebellar nuclei can be driven to fire at >100 Hz in slice preparations (Bagnall et al., 2009). Therefore, as an initial step we examined the effect of high frequency FN stimulation (100 Hz, 100 stimuli, 1 s duration, 100 μA) on PrL cell firing rates in the awake, behaving rat. Recordings were made from 20 cells in 3 animals as they sat quietly on an elevated rest platform (see methods for further details) whilst the contralateral FN was stimulated. Of the cells recorded, 50% (10 cells) displayed a decreased firing rate compared to baseline activity (mean baseline firing rate = 5.8 ± 1.2 Hz, see Table 1), 5% (1 cell) showed a significant increase, whereas 40% (8 cells) displayed a biphasic response (see Figure 3). Only 5% of the sample (1 cell) exhibited no change in firing rate following stimulation.

**Table 1:**
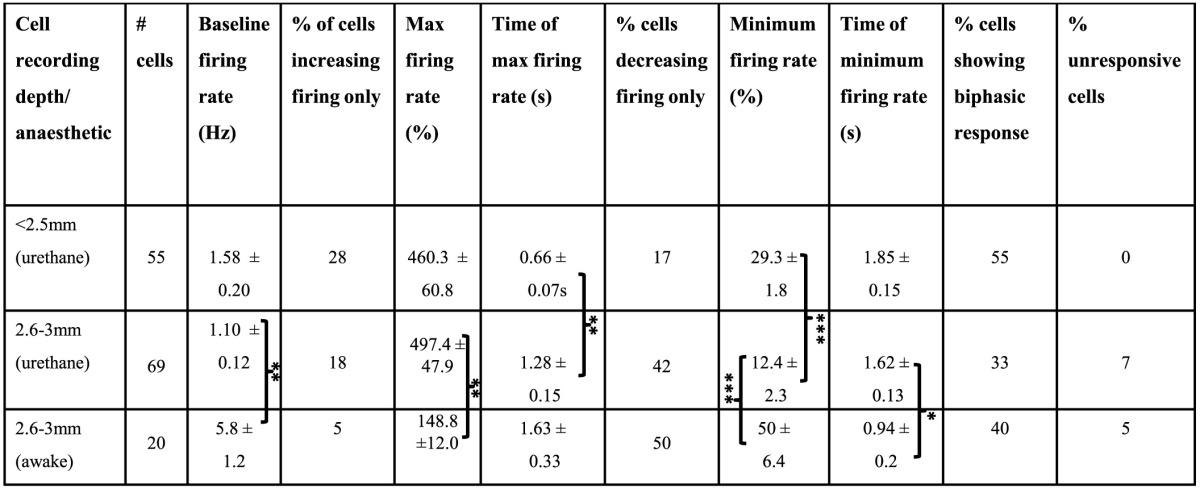
**Frontal cortex cell responses following FN stimulation**.

*P-values between data pairs marked by brackets. One-Way ANOVA with Tukey's multiple comparison test between cells recorded at <2.5 mm depth and those at 2.6–3 mm under anesthesia, and cells recorded between 2.6 and 3 mm in anesthesia and awake states*.

^*^P < 0.05; ^**^P < 0.01; ^***^P < 0.001.

Consistently rhythmic cell firing was not detected in the awake animal, and FN stimulation did not modulate either auto- or cross-correlations (data not shown), most likely due to stochastic, behavior-dependent PrL firing in the awake, behaving rat (Jung et al., 1998). Therefore, in order to examine the influence of cerebellar stimulation on more stationary frontal cortical firing, we next examined the result of FN stimulation on putative pyramidal cell firing rates in rats anesthetized with urethane, which induces dominant slow-wave oscillations (cf. Clement et al., 2008) that have been suggested to play an important role in neocortical-cerebellar communication (Ros et al., 2009; Rowland et al., 2010).

Using the same parameters as for awake rats (100 Hz, 100 stimuli, 1 s duration, 100 μA), we found that stimulation of the contralateral FN resulted in a robust but heterogeneous modulation of frontal cortex putative pyramidal cell firing (see Figure 3 and Table 1 for comparison of response characteristics). From a total of 55 cells recorded (*n* = 5 rats) in M2 and Cg1 regions of frontal cortex under urethane anesthesia, 27.3% (15 cells) displayed a significant increase in firing rate compared to pre-stimulation baseline activity, whereas 16.4% (9 cells) showed a significant firing rate decrease. A third category of cells (56.3%; 31 cells) showed a biphasic response that generally consisted of a significant firing rate decrease and subsequent increase (see Table 1). Cells recorded from tetrodes positioned at depths corresponding to PrL (*n* = 8 rats; 69 cells) were also heterogeneously modulated by the FN stimulation but the overall pattern of responses observed in PrL cells was significantly different to those recorded in M2/Cg1 (PrL cells: 12/69 (17.4%) displayed an increase in firing rate, 29/69 (42 %) displayed a decrease in firing rate, 23/69 (33.3%) showed a biphasic pattern, and 5/69 (7.3%) showed no response (Figure 3C, when the proportion of cells in these different categories were compared to those found in M2/Cg1 χ^2^ = 15.66, *df* = 3, ^**^P < 0.01; see Table 1).

Of the cells that responded to FN stimulation with decreases in firing rates, PrL cells showed more profound firing rate reductions than M2/Cg1 cells (to 29.3 ± 1.8% and 12.4 ± 2.3% of baseline respectively; ^***^P < 0.001, One-Way ANOVA with Tukey's multiple comparison test; see Table 1). Of cells responding with increased firing rates, relative increases were similar in PrL and M2/Cg1 populations (to 497 ± 47.9% and 460 ± 60.8% respectively), though FN stimulation-induced firing peaked more rapidly in M2/Cg1 than in PrL (0.66 ± 0.07 s following stimulation compared to 1.28 ± 0.15 s ^**^P < 0.01, One-Way ANOVA with Tukey's multiple comparison test). Overall, these data therefore reflect a complex pattern of modulation, with M2/Cg1 cells tending to respond with a rapid biphasic response and PrL cells typically displaying an initial reduction in firing rate following cerebellar stimulation.

This overall pattern of response did not differ significantly from the equivalent recordings made in awake rats (χ^2^= 2.2, *df* = 3, *P* > 0.05; see Table 1). However, compared to recordings made in awake rats, PrL cell firing rate increases/decreases in anesthetized animals were significantly more pronounced following FN stimulation (See Figure 3 and Table 1).

**Figure 4:**
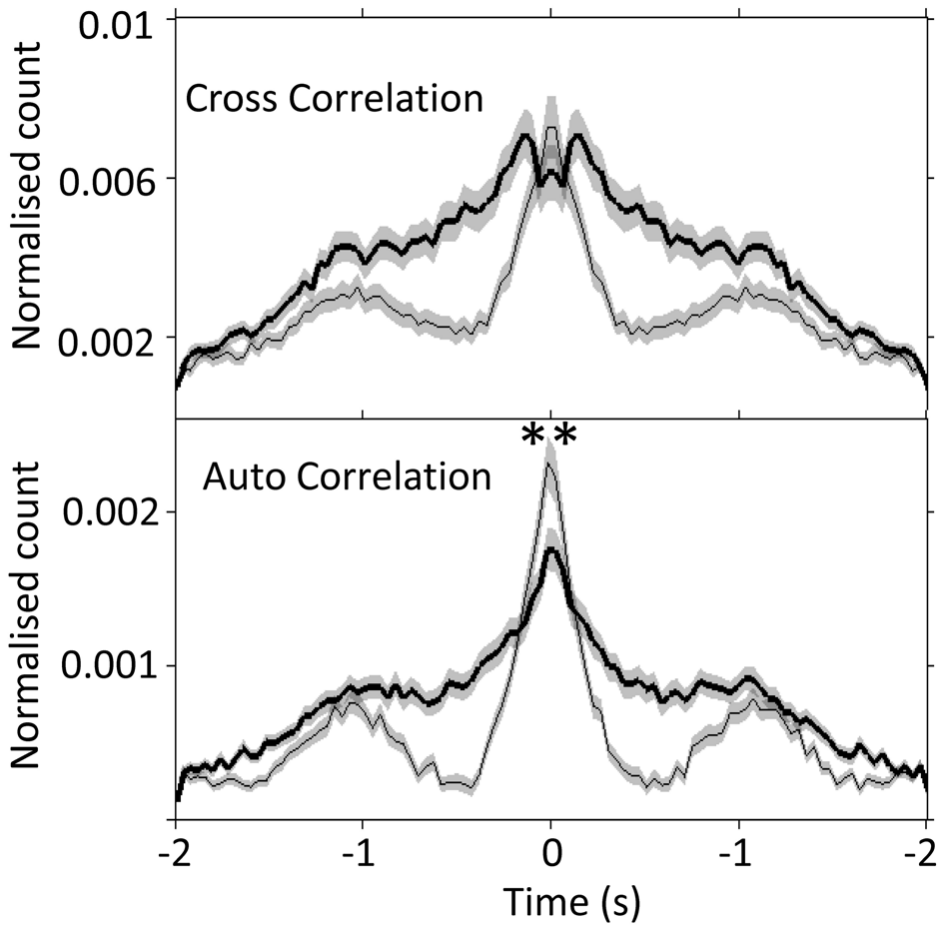
**Slow-wave modulation of PrL activity is disrupted by FN stimulation in urethane anesthetized rats**. Cross- and auto-correlogram plots (40 ms bins, calculated over 2 s pre/post-stimulation) of all possible PrL cell pair combinations (*n* = 69 cells, 8 rats) during non-stimulated (thin line) and FN stimulation (thick black line) states in urethane anesthetized rats. ^**^P < 0.01, paired *t*-test. Note attenuation of slow-wave periodicity during FN stimulation also reported in anesthetized cat (Steriade, 1995).

Next, by using auto- and cross- correlogram analyses, we investigated the effect of FN stimulation on the average, coordinated network rhythmicity within the PrL in urethane anesthetized rats. Despite the heterogeneity in PrL cell responses shown in Figure 3 and Table 1, cerebellar stimulation resulted in modulation of ongoing population PrL network activity, as observed in the disruption of slow wave oscillations, and broadening of the central peak in both auto- and cross-correlations (see Figure 4). This finding highlights the potential of the cerebellum to influence ongoing network processing in the neocortex and provides further evidence of functional connectivity between the regions.

### Cerebello- prefrontal communication in awake rats

As a first step to understanding cerebello-prefrontal network activity and interactions in behaving animals, we examined the covariance of prelimbic cortical and fastigial nucleus LFP signals using Fourier coherence analysis (see methods for further details) during active locomotion in a 1 m diameter open field arena vs. quiet restfulness on a 20 cm platform.

**Figure 5:**
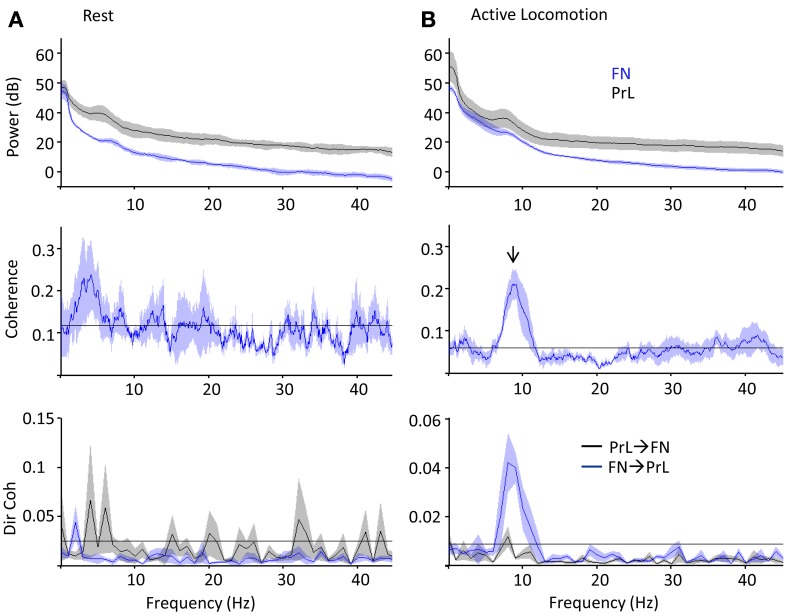
**PrL-FN LFP network activity during rest and open field exploration**. Grouped power spectra (top panel; cerebellum/FN in blue, prelimbic cortex/PrL in black), coherence (middle panel) and directed coherence (Dir coh, bottom panel; FN-PrL direction in blue, PrL-FN in black) as rats sat quietly on rest platform (**A**, *n* = 5) or actively moved in the open field arena (**B**, 1 Hz bandwidth; *n* = 4). Confidence level at *P* = 0.05 marked by horizontal black lines, shading indicates jack-knife error bars. Arrow indicates significant coherence peak at theta frequency, which was only during active locomotion and preferentially driven in the FN-PrL direction.

Cerebellar theta power (5–10 Hz) showed a slight increase during active locomotion relative to rest (Figure 5A top; FN theta power during rest, 17 ± 1.6 dB; active locomotion, 25 ± 2.3 dB; *P* < 0.05, Wilcoxon rank sum test; *n* = 4 for open field and 5 for rest box recordings), whilst PrL theta power was similar in the two behavioral states (PrL theta during rest, 30 ± 4.8 dB; active locomotion, 29 ± 6.0 dB; *P* > 0.05). Despite these limited power changes, the FN LFP signal was significantly and selectively coherent with PrL oscillations in the theta range (5–10 Hz) only during active locomotion in the open field (*P* < 0.05, arrow in Figure 5B), but not while rats were at rest (Figure 5A). Consequently, the proportion of total coherence carried at theta frequency (a ratio between 5–10 Hz coherence and coherence at all other frequencies up to 45 Hz) was significantly higher during locomotion (ratio 1.85 ± 0.21) than rest (0.97 ± 0.01; *P* < 0.05, Wilcoxon rank sum).

Since coherence is a measure of consistent phase relationships and does not quantify the direction of interaction between two signals, we also used Granger causality (directed coherence; see methods for details) to infer directionality from the simultaneous FN and PrL LFP recordings (Figure 5). Uni-directional theta coherence was significantly weighted in the FN-PrL direction when animals were actively moving in the open field (see Figure 5B lower panel; *P* < 0.05 FN-PrL vs. PrL-FN, Wilcoxon rank sum test; *n* = 4). In contrast, theta coherence was no longer significantly directional (FN-PrL and PrL-FN comparision *P* > 0.05) and was significantly lower during rest (*P* < 0.05 vs. active locomotion, Wilcoxon rank sum test).

These coherence analyses provide evidence to suggest that the cerebellar-prefrontal connectivity exemplified by FN stimulation-evoked responses in PFC could subserve cross-structural, network interactions that preferentially manifest at theta frequencies during active behavior.

## Discussion

The key findings of the current study are that: (1) stimulation of the FN evokes short latency (~13 ms) field potentials in PrL alongside changes in frontal cortical neuronal firing rates and rhythmicity and (2) FN and PrL LFP are coherent in the theta (5–10 Hz) frequency range during active locomotion, an effect preferentially driven in the FN-PrL direction. These findings demonstrate physiological interactions between vermal cerebellum and prelimbic cortex in rat and provide insights into the neural dynamics of the reciprocally connected networks underpinning cerebellar-cerebro communication.

### Cerebellar-prefrontal connectivity

Recent physiological evidence from rats indicates the presence of a prefrontal-olivo-cerebellar projection specifically to vermal lobule VII (Watson et al., 2009). In the current study we targeted the fastigial nucleus, the output of vermal lobule VII known to integrate signals from the cerebellar cortical A-zone (e.g., Voogd and Ruigrok, 2004), to examine reciprocal cerebellofugal influence on the prefrontal cortex. Our finding that fastigial stimulation can evoke discrete field potentials in the PrL (Figure 2) corroborates previous electrophysiological studies: fastigial nucleus projects to widespread cerebral cortical areas via the ventromedial thalamic nuclei in cat (~10 ms latency; Steriade, 1995), plus cerebellar dentate nucleus stimulation results in short latency field potential responses in the prefrontal association areas of monkeys (2–4.5 ms latency; Sasaki et al., 1979).

Although we cannot categorically rule out incidental stimulation of neighboring cerebellar nuclei, our histological verification of stimulation sites, inter-animal consistency of LFP and comparable results in cat lend weight to the fastigial nucleus constituting a key relay in cerebello-cerebral interactions. The latency observed in the current study (~13 ms) suggests that the cerebello-prefrontal pathway in rat is relatively slow conducting and/or polysynaptic, presumably involving at least one synaptic relay (most likely within the thalamus), though it is possible that a direct pathway to a frontal subregion other than M2/Cg1/PrL does exist. Alternatively, the shorter latency and faster conduction of the cerebello-prefrontal pathway in non-human primate could reflect evolution of a more rapid, direct line of communication in line with the increasing importance of this pathway in cerebellar contributions to cognitive functions (Ramnani, 2006).

### Cerebellar modulation of prefrontal firing

Electrical stimulation of the fastigial nuclei drives heterogeneous modulation of prefrontal cell firing in both anesthetized and behaving rats. Preliminary findings in awake mice have reported similar frontal responses to cerebellar cortical stimulation, showing reductions in synchronized firing and pauses in cortical cell firing (Liu et al., 2012). Steriade (1995) showed that fastigial stimulation can modulate gamma frequency (~20–40 Hz) EEG rhythms in the frontal cortex of anesthetized cats (with effects outlasting the duration of stimulus by ~4–5 s) and cerebellar cortical stimulation has recently been shown to modulate EEG activity recorded over the motor cortex of awake mice (M1, Witter et al., 2013), thus highlighting the presence of multiple cerebello-cortical pathways.

Mittleman et al. (2008) described long lasting (7–8 s) dopamine efflux in the PrL of mice following cerebellar dentate nuclear stimulation and dopamine release may contribute to the effects on PrL found in the present experiments. Primate dorsolateral prefrontal cortex pyramidal neurons express mixed dopamine receptor distributions (Lidow et al., 1991; Muly et al., 1998), and *in vitro* experiments have highlighted the opposing effects that D1 and D2 receptor types exert on prefrontal neuron spiking (Seamans et al., 2001). The heterogeneous effects of fastigial nuclear stimulation on individual pyramidal cell responses summarized in Table 1 may therefore reflect mixed dopamine receptor subtype expression and/or targeting by dopaminergic afferents.

The pathway(s) through which cerebellar stimulation can influence prefrontal dopamine release are unknown but one potential route includes projections via the thalamic nuclei, which send glutamatergic afferents to the cerebral cortex (Hoover and Vertes, 2007) that in turn form presynaptic inputs to dopamine varicosities within the prefrontal cortex (leading to a slow, neuromodulatory response, Blaha et al., 1997). Alternatively, fastigial stimulation could influence the release of dopamine in the prefrontal cortex via activation of the ventral tegmental area (VTA) through cerebello-VTA or cerebello-thalamo-VTA projections (Kehr et al., 1976; Snider and Maiti, 1976; Snider et al., 1976). The cerebellum may also influence prefrontal firing via the basal ganglia. For example, electrical stimulation of the cerebellar output nuclei (dentate in particular) alters neuronal firing rates (Li and Parker, 1969; Ratcheson and Li, 1969) and dopamine levels in the substantia nigra and caudate nucleus (Nieoullon et al., 1978). Anatomical studies demonstrate disynaptic projections from the cerebellum (dentate) to the basal ganglia in both rat and monkey (Ichinohe et al., 2000; Hoshi et al., 2005). In turn, basal ganglia projections influence prefrontal cortex (e.g., Middleton and Strick, 1994; Maurice et al., 1999; Middleton and Strick, 2002), thus highlighting the basal ganglia as a potential relay between hind- and forebrain.

### Coherent prefrontal-cerebellar LFP during behavior

Neurobiological oscillations organize activity within and across different brain regions (e.g., Singer, 1999; Varela et al., 2001; Fries, 2005), creating coherent cell assemblies (Harris et al., 2003) and enabling plasticity processes dependent on the precise timing of pre- and post-synaptic activity (Markram et al., 1997; Bi and Poo, 1998; Cassenaer and Laurent, 2007). Coordinated oscillations may therefore support information transfer in cerebro-cerebellar pathways and have been reported across a range of frequencies: cerebellar oscillations phase-lock to neocortical slow waves (0–4 Hz; Ros et al., 2009; Rowland et al., 2010; Schwarz, 2010) and beta oscillations (~10–25 Hz) bind cerebellar cortical LFP and nuclear cell firing with the somatosensory and motor cortices of primates (Courtemanche and Lamarre, 2005; Soteropoulos and Baker, 2006).

Of particular relevance to the current study are (1) the demonstration by Steriade (1995) that stimulation of cat FN disrupts slow wave activity, a result corroborated by our findings (Figure 2) and (2) the additional finding that cerebellar circuits have been shown to support oscillation frequencies within the theta bandwidth (Hartmann and Bower, 1998; D'Angelo et al., 2001; Solinas et al., 2007; Dugué et al., 2009), which could be driven by pacemaker theta rhythmicity within precerebellar nuclei including the inferior olive (Lang et al., 2006; Chorev et al., 2007; Van Der Giessen et al., 2008). Theta rhythmicity in the cerebellar fastigial nuclei may therefore reflect synchronous activation or inhibition in either the olivo-nuclear or olivo-cerebello-nuclear circuitry, and there is evidence that such oscillations synchronize activity within cerebellar hemispheres as well as between cerebellar and cortical/limbic regions (Hartmann and Bower, 1998; O'Connor et al., 2002; Hoffmann and Berry, 2009; Wikgren et al., 2010).

O'Connor et al. (2002) found that LFP activity in whisker-related areas of rat cerebellar cortex and neocortex are coherent at 5–20 Hz during periods of active whisking, and whisker- and eye-movement related activity is found in the rat prefrontal region (Brecht et al., 2004). Coherent cerebellar-cerebro activity may therefore reflect mechanisms through which sensory information can be integrated into ongoing neocortical processes (cf. Bland and Oddie, 2001; O'Connor et al., 2002; Bland, 2004). Our finding that coherence is significantly weighted in the cerebellum-to-PrL direction during epochs of active locomotion (Figure 5B) suggests the interaction derives from more than simple co-modulation of cerebellum and PrL during theta-frequency behaviors (e.g., whisking). The directed nature of this coupling may reflect the increased need for sensorimotor input to the neocortex during goal-directed behaviors including active locomotion. In particular, as vestibular information is combined with proprioceptive inputs in the FN to generate appropriate internal estimates of the animal's self motion (Brooks and Cullen, 2009), the cerebellum (FN in particular) may provide functionally relevant proprioceptive/egocentric information that can be integrated into decision making processes recruiting higher order structures such as the PrL.

## Concluding comments

Cerebellar vermal abnormalities are found in a host of psychiatric diseases (Heath et al., 1979, 1982; Okugawa et al., 2007; Lawyer et al., 2009) and can occur concomitantly with changes in the prefrontal cortex of autistic patients (Carper and Courchesne, 2000). Also, chronic cerebellar vermal stimulation in the theta frequency range has been reported to ameliorate the emotional and cognitive symptoms of intractable neurological disorders such as schizophrenia and epilepsy (Cooper, 1973; Cooper et al., 1976; Correa et al., 1980). Abnormalities of the cerebellum, and particularly its vermal region, may therefore contribute to neuro-psychiatric diseases that are typically associated with neocortical malfunction and aberrant dopamine neuromodulation. The present results provide evidence for a physiological framework whose dysfunction could underlie cerebellar contributions to such disorders.

Further work monitoring and manipulating cerebello-cerebral network activity with higher resolution during a range of behavioral states (e.g., with and without explicit cognitive load) is required before we fully appreciate the functional importance of activity in cerebello-prefrontal circuits. However, the current study provides initial evidence that the regions co-participate in distributed network activity and also offers novel insights into the dynamic interaction that occurs between the two structures during exploratory behavior. The potential mechanisms subserving these interactions include synchronized oscillations in the theta frequency, which may be important for sensory acquisition/integration (Bower, 1997; Bland and Oddie, 2001) and/or general cerebellar contributions to goal directed behaviors (Burguière et al., 2010).

## Author contributions

T. C. Watson, Richard Apps, and Matthew W. Jones designed research; T. C. Watson performed research; T. C. Watson, N. Becker, and Matthew W. Jones analyzed data; T. C. Watson, Richard Apps, and Matthew W. Jones. wrote the paper.

### Conflict of interest statement

The authors declare that the research was conducted in the absence of any commercial or financial relationships that could be construed as a potential conflict of interest.

## References

[B1] Allen G. McColl R. Barnard H. Ringe W. K. Fleckenstein J. Cullum C. M. (2005). Magnetic resonance imaging of cerebellar-prefrontal and cerebellar-parietal functional connectivity. Neuroimage 28, 39–48 10.1016/j.neuroimage.2005.06.013 16023375

[B2] Andreasen N. C. O'Leary D. S. Cizadlo T. Arndt S. Rezai K. Ponto L. L. (1996). Schizophrenia and cognitive dysmetria: a positron-emission tomography study of dysfunctional prefrontal-thalamic-cerebellar circuitry. Proc. Natl. Acad. Sci. U.S.A. 93, 9985–9990 10.1073/pnas.93.18.9985 8790444 PMC38542

[B3] Andreasen N. C. Pierson R. (2008). The role of the cerebellum in schizophrenia. Biol. Psychiatry 64, 81–88 10.1016/j.biopsych.2008.01.003 18395701 PMC3175494

[B4] Arguello P. A. Enquist L. W. Wang S. S.-H. (2012). Long-distance connectivity between prefrontal cortex and cerebellum in mouse, in Society for Neuroscience (New Orleans).

[B5] Bagnall M. W. Zingg B. Sakatos A. Moghadam S. H. Zeilhofer H. U. du Lac S. (2009). Glycinergic projection neurons of the cerebellum. J. Neurosci. 29, 10104–10110 10.1523/JNEUROSCI.2087-09.2009 19675244 PMC3196611

[B6] Baker S. N. Chiu M. Fetz E. E. (2006). Afferent encoding of central oscillations in the monkey arm. J. Neurophysiol. 95, 3904–3910 10.1152/jn.01106.2005 16709725

[B7] Bi G. Q. Poo M. M. (1998). Synaptic modifications in cultured hippocampal neurons: dependence on spike timing, synaptic strength, and postsynaptic cell type. J. Neurosci. 18, 10464–10472 9852584 10.1523/JNEUROSCI.18-24-10464.1998 PMC6793365

[B8] Blaha C. D. Yang C. R. Floresco S. B. Barr A. M. Phillips A. G. (1997). Stimulation of the ventral subiculum of the hippocampus evokes glutamate receptor-mediated changes in dopamine efflux in the rat nucleus accumbens. Eur. J. Neurosci. 9, 902–911 10.1111/j.1460-9568.1997.tb01441.x 9182943

[B9] Bland B. H. (2004). The power of theta: providing insights into the role of the hippocampal formation in sensorimotor integration. Hippocampus 14, 537–538 10.1002/hipo.20027 15301432

[B10] Bland B. H. Oddie S. D. (2001). Theta band oscillation and synchrony in the hippocampal formation and associated structures: the case for its role in sensorimotor integration. Behav. Brain Res. 127, 119–136 10.1016/S0166-4328(01)00358-8 11718888

[B11] Bokil H. Andrews P. Kulkarni J. E. Mehta S. Mitra P. P. (2010). Chronux: a platform for analyzing neural signals. J. Neurosci. Methods 192, 146–151 10.1016/j.jneumeth.2010.06.020 20637804 PMC2934871

[B12] Bower J. M. (1997). Is the cerebellum sensory for motor's sake, or motor for sensory's sake: the view from the whiskers of a rat? Prog. Brain Res. 114, 463–496 10.1016/S0079-6123(08)63381-6 9193161

[B13] Brecht M. Krauss A. Muhammad S. Sinai-Esfahani L. Bellanca S. Margrie T. W. (2004). Organization of rat vibrissa motor cortex and adjacent areas according to cytoarchitectonics, microstimulation, and intracellular stimulation of identified cells. J. Comp. Neurol. 479, 360–373 10.1002/cne.20306 15514982

[B14] Brooks J. X. Cullen K. E. (2009). Multimodal integration in rostral fastigial nucleus provides an estimate of body movement. J. Neurosci. 29, 10499–10511 10.1523/JNEUROSCI.1937-09.2009 19710303 PMC3311469

[B15] Buckner R. L. Krienen F. M. Castellanos A. Diaz J. C. Yeo B. T. (2011). The organization of the human cerebellum estimated by intrinsic functional connectivity. J. Neurophysiol. 106, 2322–2345 10.1152/jn.00339.2011 21795627 PMC3214121

[B16] Burguière E. Arabo A. Jarlier F. De Zeeuw C. I. Rondi-Reig L. (2010). Role of the cerebellar cortex in conditioned goal-directed behavior. J. Neurosci. 30, 13265–13271 10.1523/JNEUROSCI.2190-10.2010 20926652 PMC6634747

[B17] Carper R. A. Courchesne E. (2000). Inverse correlation between frontal lobe and cerebellum sizes in children with autism. Brain 123(Pt 4), 836–844 10.1093/brain/123.4.836 10734014

[B18] Cassenaer S. Laurent G. (2007). Hebbian STDP in mushroom bodies facilitates the synchronous flow of olfactory information in locusts. Nature 448, 709–713 10.1038/nature05973 17581587

[B19] Chorev E. Yarom Y. Lampl I. (2007). Rhythmic episodes of subthreshold membrane potential oscillations in the rat inferior olive nuclei *in vivo*. J. Neurosci. 27, 5043–5052 10.1523/JNEUROSCI.5187-06.2007 17494690 PMC6672369

[B20] Clement E. A. Richard A. Thwaites M. Ailon J. Peters S. Dickson C. T. (2008). Cyclic and sleep-like spontaneous alternations of brain state under urethane anaesthesia. PLoS ONE 3:e2004 10.1371/journal.pone.0002004 18414674 PMC2289875

[B21] Cooper I. S. (1973). Effect of chronic stimulation of anterior cerebellum on neurological disease. Lancet 1, 206 10.1016/S0140-6736(73)90042-1 4118825

[B22] Cooper I. S. Amin I. Riklan M. Waltz J. M. Poon T. P. (1976). Chronic cerebellar stimulation in epilepsy. Clinical and anatomical studies. Arch. Neurol. 33, 559–570 10.1001/archneur.1976.00500080037006 821458

[B23] Correa A. J. Llewellyn R. C. Epps J. Jarrott D. Eiswirth C. Heath R. G. (1980). Chronic cerebellar stimulation in the modulation of behavior. Acta Neurol. Latinoam. 26, 143–153 6807046

[B24] Courtemanche R. Lamarre Y. (2005). Local field potential oscillations in primate cerebellar cortex: synchronization with cerebral cortex during active and passive expectancy. J. Neurophysiol. 93, 2039–2052 10.1152/jn.00080.2004 15590736

[B25] D'Angelo E. Nieus T. Maffei A. Armano S. Rossi P. Taglietti V. (2001). Theta-frequency bursting and resonance in cerebellar granule cells: experimental evidence and modeling of a slow k+-dependent mechanism. J. Neurosci. 21, 759–770 11157062 10.1523/JNEUROSCI.21-03-00759.2001 PMC6762330

[B26] Demirtas-Tatlidede A. Freitas C. Cromer J. R. Safar L. Ongur D. Stone W. S. (2010). Safety and proof of principle study of cerebellar vermal theta burst stimulation in refractory schizophrenia. Schizophr. Res. 124, 91–100 10.1016/j.schres.2010.08.015 20817483 PMC3268232

[B27] Dugué G. P. Brunel N. Hakim V. Schwartz E. Chat M. Lévesque M. (2009). Electrical coupling mediates tunable low-frequency oscillations and resonance in the cerebellar Golgi cell network. Neuron 61, 126–139 10.1016/j.neuron.2008.11.028 19146818

[B28] Fatemi S. H. Aldinger K. A. Ashwood P. Bauman M. L. Blaha C. D. Blatt G. J. (2012). Consensus paper: pathological role of the cerebellum in autism. Cerebellum 11, 777–807 10.1007/s12311-012-0355-9 22370873 PMC3677555

[B29] Fries P. (2005). A mechanism for cognitive dynamics: neuronal communication through neuronal coherence. Trends Cogn. Sci. 9, 474–480 10.1016/j.tics.2005.08.011 16150631

[B30] Gray C. M. (1994). Synchronous oscillations in neuronal systems: mechanisms and functions. J. Comput. Neurosci. 1, 11–38 10.1007/BF00962716 8792223

[B31] Hagan M. A. Dean H. L. Pesaran B. (2012). Spike-field activity in parietal area LIP during coordinated reach and saccade movements. J. Neurophysiol. 107, 1275–90 10.1152/jn.00867.2011 22157119 PMC3311693

[B32] Harris K. D. Csicsvari J. Hirase H. Dragoi G. Buzsáki G. (2003). Organization of cell assemblies in the hippocampus. Nature 424, 552–556 10.1038/nature01834 12891358

[B33] Harris K. D. Hirase H. Leinekugel X. Henze D. A. Buzsáki G. (2001). Temporal interaction between single spikes and complex spike bursts in hippocampal pyramidal cells. Neuron 32, 141–149 10.1016/S0896-6273(01)00447-0 11604145

[B34] Hartmann M. J. Bower J. M. (1998). Oscillatory activity in the cerebellar hemispheres of unrestrained rats. J. Neurophysiol. 80, 1598–1604 9744967 10.1152/jn.1998.80.3.1598

[B35] Heath R. G. (1977). Modulation of emotion with a brain pacemamer. Treatment for intractable psychiatric illness. J. Nerv. Ment. Dis. 165, 300–317 10.1097/00005053-197711000-00002 303280

[B36] Heath R. G. Franklin D. E. Shraberg D. (1979). Gross pathology of the cerebellum in patients diagnosed and treated as functional psychiatric disorders. J. Nerv. Ment. Dis. 167, 585–592 10.1097/00005053-197910000-00001 573778

[B37] Heath R. G. Franklin D. E. Walker C. F. Keating J. W. (1982). Cerebellar vermal atrophy in psychiatric patients. Biol. Psychiatry 17, 569–583 7093393

[B38] Henze R. Brunner R. Thiemann U. Parzer P. Richterich A. Essig M. (2011). Gray matter alterations in first-admission adolescents with schizophrenia. J. Neuroimaging 21, 241–246 10.1111/j.1552-6569.2010.00504.x 20572905

[B39] Hoffmann L. C. Berry S. D. (2009). Cerebellar theta oscillations are synchronized during hippocampal theta-contingent trace conditioning. Proc. Natl. Acad. Sci. U.S.A. 106, 21371–21376 10.1073/pnas.0908403106 19940240 PMC2795537

[B40] Hoover W. B. Vertes R. P. (2007). Anatomical analysis of afferent projections to the medial prefrontal cortex in the rat. Brain Struct. Funct. 212, 149–179 10.1007/s00429-007-0150-4 17717690

[B41] Hoshi E. Tremblay L. Feger J. Carras P. L. Strick P. L. (2005). The cerebellum communicates with the basal ganglia. Nat. Neurosci. 8, 1491–1493 10.1038/nn1544 16205719

[B42] Ichinohe N. Mori F. Shoumura K. (2000). A di-synaptic projection from the lateral cerebellar nucleus to the laterodorsal part of the striatum via the central lateral nucleus of the thalamus in the rat. Brain Res. 880, 191–197 10.1016/S0006-8993(00)02744-X 11033006

[B43] Jung M. W. Qin Y. McNaughton B. L. Barnes C. A. (1998). Firing characteristics of deep layer neurons in prefrontal cortex in rats performing spatial working memory tasks. Cereb. Cortex 8, 437–450 10.1093/cercor/8.5.437 9722087

[B44] Kehr W. Lindqvist M. Carlsson A. (1976). Distribution of dopamine in the rat cerebral cortex. J. Neural Transm. 38, 173–180 10.1007/BF01249437 956808

[B45] Kelly R. M. Strick P. L. (2003). Cerebellar loops with motor cortex and prefrontal cortex of a nonhuman primate. J. Neurosci. 23, 8432–8444 12968006 10.1523/JNEUROSCI.23-23-08432.2003 PMC6740694

[B46] Krienen F. M. Buckner R. L. (2009). Segregated fronto-cerebellar circuits revealed by intrinsic functional connectivity. Cereb. Cortex 19, 2485–2497 10.1093/cercor/bhp135 19592571 PMC2742600

[B47] Lang E. J. Sugihara I. Llinás R. (2006). Olivocerebellar modulation of motor cortex ability to generate vibrissal movements in rat. J. Physiol. 571, 101–120 10.1113/jphysiol.2005.102764 16357010 PMC1805652

[B48] Lawyer G. Nesvåg R. Varnäs K. Okugawa G. Agartz I. (2009). Grey and white matter proportional relationships in the cerebellar vermis altered in schizophrenia. Cerebellum 8, 52–60 10.1007/s12311-008-0071-7 18972181

[B49] Li C. L. Parker L. O. (1969). Effect of dentate stimulation on neuronal activity in the globus pallidus. Exp. Neurol. 24, 298–309 10.1016/0014-4886(69)90023-5 5784137

[B50] Lidow M. S. Goldman-Rakic P. S. Gallager D. W. Rakic P. (1991). Distribution of dopaminergic receptors in the primate cerebral cortex: quantitative autoradiographic analysis using [3H]raclopride, [3H]spiperone and [3H]SCH23390. Neuroscience 40, 657–671 10.1016/0306-4522(91)90003-7 2062437

[B51] Liu Y. Blaha C. D. Mittleman G. Goldowitz D. Heck D. H. (2012). Cerebellar modulation of neuronal activity in mouse prefrontal cortex, in Society For Neuroscience (New Orleans).

[B52] Markram H. Lübke J. Frotscher M. Sakmann B. (1997). Regulation of synaptic efficacy by coincidence of postsynaptic APs and EPSPs. Science 275, 213–215 10.1126/science.275.5297.213 8985014

[B53] Maurice N. Deniau J. M. Glowinski J. Thierry A. M. (1999). Relationships between the prefrontal cortex and the basal ganglia in the rat: physiology of the cortico-nigral circuits. J. Neurosci. 19, 4674–81 10341265 10.1523/JNEUROSCI.19-11-04674.1999 PMC6782607

[B54] Middleton F. A. Strick P. L. (2002). Basal ganglia ‘projections’ to the prefrontal cortex of the primate. Cereb. Cortex. 12, 926–35 10.1093/cercor/12.9.926 12183392

[B55] Middleton F. A. Strick P. L. (1994). Anatomical evidence for cerebellar and basal ganglia involvement in higher cognitive function. Science 266, 456–61 10.1126/science.7939688 7939688

[B56] Middleton F. A. Strick P. L. (2001). Cerebellar projections to the prefrontal cortex of the primate. J. Neurosci. 21, 700–712 11160449 10.1523/JNEUROSCI.21-02-00700.2001 PMC6763818

[B57] Mittleman G. Goldowitz D. Heck D. H. Blaha C. D. (2008). Cerebellar modulation of frontal cortex dopamine efflux in mice: relevance to autism and schizophrenia. Synapse 62, 544–550 10.1002/syn.20525 18435424 PMC3854870

[B58] Muly E. C. Szigeti K. Goldman-Rakic P. S. (1998). D1 receptor in interneurons of macaque prefrontal cortex: distribution and subcellular localization. J. Neurosci. 18, 10553–10565 9852592 10.1523/JNEUROSCI.18-24-10553.1998 PMC6793362

[B59] Nieoullon A. Cheramy A. Glowinski J. (1978). Release of dopamine in both caudate nuclei and both substantia nigrae in response to unilateral stimulation of cerebellar nuclei in the cat. Brain Res. 148, 143–152 10.1016/0006-8993(78)90384-0 656921

[B60] O'Connor S. M. Berg R. W. Kleinfeld D. (2002). Coherent electrical activity between vibrissa sensory areas of cerebellum and neocortex is enhanced during free whisking. J. Neurophysiol. 87, 2137–2148 10.1152/jn.00229.2001 11929931

[B61] Okugawa G. Nobuhara K. Takase K. Kinoshita T. (2007). Cerebellar posterior superior vermis and cognitive cluster scores in drug-naive patients with first-episode schizophrenia. Neuropsychobiology 56, 216–219 10.1159/000122268 18382120

[B62] O'Reilly J. X. Beckmann C. F. Tomassini V. Ramnani N. Johansen-Berg H. (2010). Distinct and overlapping functional zones in the cerebellum defined by resting state functional connectivity. Cereb. Cortex 20, 953–965 10.1093/cercor/bhp157 19684249 PMC2837094

[B63] Paxinos G. Watson C. (2006). The Rat Brain in Stereotaxic Coordinates, 6th Edn. New York, NY: Academic

[B64] Ramnani N. (2006). The primate cortico-cerebellar system: anatomy and function. Nat. Rev. Neurosci. 7, 511–522 10.1038/nrn1953 16791141

[B65] Ratcheson R. A. Li C. L. (1969). Effect of dentate stimulation on neuronal activity in the caudate nucleus. Exp. Neurol. 25, 268–281 10.1016/0014-4886(69)90050-8 5345013

[B66] Rochefort C. Arabo A. André M. Poucet B. Save E. Rondi-Reig L. (2011). Cerebellum shapes hippocampal spatial code. Science 334, 385–389 10.1126/science.1207403 22021859

[B67] Ros H. Sachdev R. N. Yu Y. Sestan N. McCormick D. A. (2009). Neocortical networks entrain neuronal circuits in cerebellar cortex. J. Neurosci. 29, 10309–10320 10.1523/JNEUROSCI.2327-09.2009 19692605 PMC3137973

[B68] Rowland N. C. Goldberg J. A. Jaeger D. (2010). Cortico-cerebellar coherence and causal connectivity during slow-wave activity. Neuroscience 166, 698–711 10.1016/j.neuroscience.2009.12.048 20036719 PMC2823967

[B69] Sasaki K. Jinnai K. Gemba H. Hashimoto S. Mizuno N. (1979). Projection of the cerebellar dentate nucleus onto the frontal association cortex in monkeys. Exp. Brain Res. 37, 193–198 10.1007/BF01474266 114403

[B70] Schmahmann J. D. (2004). Disorders of the cerebellum: ataxia, dysmetria of thought, and the cerebellar cognitive affective syndrome. J. Neuropsychiatry Clin. Neurosci. 16, 367–378 10.1176/appi.neuropsych.16.3.367 15377747

[B71] Schwarz C. (2010). The fate of spontaneous synchronous rhythms on the cerebrocerebellar loop. Cerebellum 9, 77–87 10.1007/s12311-009-0143-3 19902318

[B72] Seamans J. K. Gorelova N. Durstewitz D. Yang C. R. (2001). Bidirectional dopamine modulation of GABAergic inhibition in prefrontal cortical pyramidal neurons. J. Neurosci. 21, 3628–3638 11331392 10.1523/JNEUROSCI.21-10-03628.2001 PMC6762481

[B73] Singer W. (1999). Neuronal synchrony: a versatile code for the definition of relations? Neuron 24, 49–65, 111–125. 10.1016/S0896-6273(00)80821-1 10677026

[B74] Snider R. S. Maiti A. (1976). Cerebellar contributions to the Papez circuit. J. Neurosci. Res. 2, 133–146 10.1002/jnr.490020204 950678

[B75] Snider R. S. Maiti A. Snider S. R. (1976). Cerebellar pathways to ventral midbrain and nigra. Exp. Neurol. 53, 714–728 10.1016/0014-4886(76)90150-3 1001395

[B76] Solinas S. Forti L. Cesana E. Mapelli J. De Schutter E. D'Angelo E. (2007). Fast-reset of pacemaking and theta-frequency resonance patterns in cerebellar golgi cells: simulations of their impact *in vivo*. Front. Cell Neurosci. 1:4 10.3389/neuro.03.004.2007 18946522 PMC2525929

[B77] Soteropoulos D. S. Baker S. N. (2006). Cortico-cerebellar coherence during a precision grip task in the monkey. J. Neurophysiol. 95, 1194–1206 10.1152/jn.00935.2005 16424458

[B78] Steriade M. (1995). Two channels in the cerebellothalamocortical system. J. Comp. Neurol. 354, 57–70 10.1002/cne.903540106 7615875

[B79] Stoodley C. J. (2012). The cerebellum and cognition: evidence from functional imaging studies. Cerebellum 11, 352–365 10.1007/s12311-011-0260-7 21373864

[B80] Stoodley C. J. Schmahmann J. D. (2010). Evidence for topographic organization in the cerebellum of motor control versus cognitive and affective processing. Cortex 46, 831–844 10.1016/j.cortex.2009.11.008 20152963 PMC2873095

[B81] Strick P. L. Dum R. P. Fiez J. A. (2009). Cerebellum and nonmotor function. Annu. Rev. Neurosci. 32, 413–434 10.1146/annurev.neuro.31.060407.125606 19555291

[B82] Suzuki L. Coulon P. Sabel-Goedknegt E. H. Ruigrok T. J. (2012). Organization of cerebral projections to identified cerebellar zones in the posterior cerebellum of the rat. J. Neurosci. 32, 10854–10869 10.1523/JNEUROSCI.0857-12.2012 22875920 PMC6621006

[B83] Van Der Giessen R. S. Koekkoek S. K. van Dorp S. De Gruijl J. R. Cupido A. Khosrovani S. (2008). Role of olivary electrical coupling in cerebellar motor learning. Neuron 58, 599–612 10.1016/j.neuron.2008.03.016 18498740

[B84] Varela F. Lachaux J. P. Rodriguez E. Martinerie J. (2001). The brainweb: phase synchronization and large-scale integration. Nat. Rev. Neurosci. 2, 229–239 10.1038/35067550 11283746

[B85] Voogd J. Ruigrok T. J. (2004). The organization of the corticonuclear and olivocerebellar climbing fiber projections to the rat cerebellar vermis: the congruence of projection zones and the zebrin pattern. J. Neurocytol. 33, 5–21 10.1023/B:NEUR.0000029645.72074.2b 15173629

[B86] Watson T. C. Jones M. W. Apps R. (2009). Electrophysiological mapping of novel prefrontal—cerebellar pathways. Front. Integr. Neurosci. 3:18 10.3389/neuro.07.018.2009 19738932 PMC2737490

[B87] Wikgren J. Nokia M. S. Penttonen M. (2010). Hippocampo-cerebellar theta band phase synchrony in rabbits. Neuroscience 165, 1538–1545 10.1016/j.neuroscience.2009.11.044 19945512

[B88] Williams E. R. Soteropoulos D. S. Baker S. N. (2009). Coherence between motor cortical activity and peripheral discontinuities during slow finger movements. J. Neurophysiol. 102, 1296–1309 10.1152/jn.90996.2008 19474171 PMC2724360

[B89] Witter L. Canto C. B. Hoogland T. M. de Gruijl J. R. De Zeeuw C. I. (2013). Strength and timing of motor responses mediated by rebound firing in the cerebellar nuclei after Purkinje cell activation. Front. Neural Circuits 7:133 10.3389/fncir.2013.00133 23970855 PMC3748751

